# A step toward autonomy in education: probing into the effects of practicing self-assessment, resilience, and creativity in task supported language learning

**DOI:** 10.1186/s40359-023-01478-8

**Published:** 2023-12-07

**Authors:** Mubarak S. Aldosari, Haroon N. Alsager

**Affiliations:** 1 Department of Special Education, Prince Sattam Bin Abdulaziz University, Al-Kharj, Saudi Arabia; 2 Department of English, Prince Sattam Bin Abdulaziz University, Al-Kharj, Saudi Arabia

**Keywords:** Autonomy, Creativity, Resilience, Self-assessment, Task supported language learning

## Abstract

Self-assessment (SA) can provide students with opportunities to self-evaluate, or make judgments about their learning process and products of learning. Regarding the importance of SA, this survey pursued to examine the effects of practicing SA on Saudi Arabian EFL learners’ resilience, creativity, and autonomy in task supported language learning. To fulfill these objectives, 60 intermediate EFL learners were chosen and separated accidentally into two groups of control and experimental. They were then pre-tested using three related questionnaires of resilience, creativity, and autonomy. Next, the treatment was practiced on the two groups. Eight lessons of Touchstone Book 3 were taught to the experimental group using SAvia applying different tasks. On the other hand, the lessons were trained to the control group without using SA and tasks. The aforementioned questionnaires were re-administered as the post-tests following the completion of all lessons. Independent and paired samples t-test findings displayed that the control and experimental groups performed differently on the three post-tests. In essence, the results showed that the experimental group's resilience, creativity, and autonomy were all improved by the treatment. The research's implications and conclusions were then outlined. The implications of the research can allow students to evaluate their own progress and skill development critically.

## Preliminaries

A learning strategy for integrating student-centered learning to promote effective autonomous learning is SA. SA can be exploited as an alternate method for students to evaluate their own efforts in order to increase student engagement [1]. By combining self-regulation, self-observation, and self-instruction, SA offers pupils a means of evaluation [2]. Additionally, it is stated that using SA throughout the evaluation process, which has a direct impact on students' academic and practical achievements, might help students enhance their metacognition and self-regulation. It demonstrates how explicitly providing student-centered learning via SA enables pupils to become more autonomous [3].

However, a student's capacity for learning autonomy also affects how they self-evaluate. According to a previous study, there is a substantial association amongst learning autonomy and SA since learning autonomy refers to students' capacities for managing their own learning, which motivates them to do so [4]. It is a lengthy description that describes SA as a component of autonomy in learning that helps pupils decide, organize, implement, and evaluate their own work [5].

Based on [6], the general approach on learning autonomy emphasizes students' power over their learning process and the learning conditions that enable autonomous learning on the basis of formal contexts. It suggests that students' learning autonomy determines how successfully they self-evaluate their work [7]. Self-evaluation is certainly constrained by learning autonomy. Many scholars are interested in the link amongst SA and learning autonomy. According to previous research, the relationship of learning autonomy and SA may be seen from the constructive feedback provided by the pupils and the usage of SA that enhances their competence [8].

SA can be done via using tasks. The principles behind task-based language instruction are that language learners may learn a language more effectively by engaging with people while doing activities and by concentrating on the message rather than the form of the language [9]. The study of second language learning in this field is growing. This method's apparent significance can be explained in several ways: In order to facilitate natural learning, it first gives students realistic real-world activities (tasks having significance and value outside of the classroom) to do. Task-based language teaching, which places a focus on the learner, sees language as a tool for communication through involving students in the normal, real, and practical usage of language for a worthwhile goal [10]. Second, because meaning is prioritized over form, learners are free to complete tasks using any language they currently know.

The goal of task-based language teaching is to maximize student engagement throughout the learning and teaching of a language. Additionally, the variety of tasks accessible (problem-solving, role-plays, surveys, reading texts, listening texts, etc.) provides a lot of flexibility in this approach and ought to result in more engaging tasks for the students [11]. Finally, activities are no longer broken down into the four language skills in the task-based approach. The four domains should be included into communication activities, according to modern techniques like TBI [9].

Using task-based language teaching can affect resilience of the students. Resilience boosts our opportunities of succeeding in schools and achieving other life goals notwithstanding challenges brought on by our original traits, circumstances, and experiences [12]. Resistance studies have gained popularity recently, and several models and datasets have been improved despite skepticism of the usefulness of such a construct in psychology. Self-efficacy empowers people to deal with problems and pressures by making them aware of their own resources and capabilities, which makes resilience a quantifiable construct [13].

As [14] stated, resilience is an active developmental procedure which encourages constructive adaptation to demanding, challenging, and stressful situations. Via resilience, people may cope with unpleasant emotions, give difficult situations a positive spin, and adjust to changing external pressures throughout their lives [15]. One of the elements that foster resilience is creativity [16, 17]. Flexibility, resourcefulness, adaptability, and originality are shared personal traits of creative and resilient people [18], as well a variety of contextual elements including community and family support [19, 20].

For handling changes, producing innovation and invention, and addressing the difficulties of our progressively intricate society, creativity, which is explained as the individuals’ potential to develop unique, adaptable, and insightful opinions and resolutions, seems to be a vital resource [21–23] stated that creativity is the capacity to produce original and useful ideas by fusing previously existing elements. Therefore, coming up with fresh ideas is what distinguishes creativity, whereas innovation also requires implementing changes based on those ideas. The capability to create original thoughts, sensible solutions, and well-thought-out strategies in response to a particular issue has been characterized as creativity in a cognitive perspective [24].

Applying task-based language teaching can affect learners’ autonomy. Since it can enhance learning, learner autonomy is recognized as an important attribute that all English language students should possess [25]. The ability to direct one's own learning and the accountability for all decisions pertaining to all facets of that learning are both instances of autonomy, according to [26]. Autonomy in language learning requires management and content control over the cognitive learning process [27]. Regardless of how LA is defined, it is crucial to remember that pupils are responsible for their own learning and participate more actively in learning. It is advised that learners create their own learning processes and tactics as well as select their own learning resources, teaching methodologies, and evaluation techniques [28].

Learner autonomy, according to [29], is a sign of a learner's maturity, self-motivation, and flexibility of learning. As [30] asserted, autonomy is the capability of a students to determine their own rate of learning and to assume ownership of what and how they learn. Researchers [31] and [32] examined how learner autonomy affected students' performance in English language learning, and they discovered that it did. These results are consistent with the theories of [33], who hypothesized that autonomy was an intrinsic potential of students that is nurtured and advanced by instructors through adopting a pertinent curriculum.

Even though their teachers are knowledgeable and qualified, [34] generally asserts that learners require learner autonomy to acquire a language. Therefore, learner autonomy is crucial to language acquisition. Just when EFL students are both extrinsically and internally motivated can learner autonomy be meaningful. [35] agreed that motivation is necessary in EFL classes in order to foster learner autonomy, if the setting in which learning takes place is learner-centered. Students have the freedom to choose the learning that is suitable for their abilities and efforts. Although the majority of learning occurs via direct experience, teachers are still needed, albeit in very small amounts. Additionally, independent learning ought not to be confused with learning without a teacher, according to [36]. When students grow independent, the teacher's job might not disappear. Because of the necessity for the teacher to serve in these increasingly demanding and open-ended capacities as a counselor, advisor, and expert.

Regarding the significant role the defined variables play in language learning and teaching, the present research aimed at examining the effects of practicing self-assessment in task supported language learning on Saudi Arabian EFL learners’ autonomy. In addition, this study intended to know if Saudi Arabian EFL learners’ creativity and resilience developed by practicing self-assessment in task supported language learning. The significance of this research can be referred to the fact that the topic under the investigation is novel and it includes three important dependent variables such as autonomy, creativity, and resilience. In fact, most previous studies examined the effects of SA on English language main skills and sub skills but the present survey tried to work on those psychological variables that play a crucial part in English language learning and teaching. Also, this study can be significant since SA is important not just for students, but will also play a role in the lives of working professionals. It aids the people reflect on their behaviors and actions and also test their knowledge effectively, which assists to understand the right course correction or to boost confidence when done right.

## Review of the literature

### Role of creativity and resilience in education

It takes work, dedication, involvement, and assessment to emphasize resilience and creative education. Its importance, application, and value cannot be limited to the utilitarian-reductionist rationality of a thing's or an idea's purpose and use since doing so would constrain the creative process and lead to conflicts [37]. The results indicate that there may be a gap in our knowledge of creativity as a vital constituent of the development of problem solving and critical thinking as well as a strategy to significantly enhance learning [38]. Higher-order thinking abilities are necessary for creativity, which is essential for learning across the board. Every subject of study has problems that necessitate creative solutions. Teachers must assist students in realizing and enhancing their creative potentiality if they are to get the inventive engineers, entrepreneurs, artists, and scientists of the future and be able to adapt to a continuously changing world [39].

By encouraging creativity and resiliency to the trauma of schools’ viciousness, the creation of practical community-oriented convergence instruction via art can encourage the growth of a jubilant school setting [40]. It is essential to understand the superseding mechanisms by which team resilience could assist the creativity of the undergraduate students in order to create policies and execute interventions in educational environments effectively. As a starting point [41], note that there aren't many empirical researches that demonstrate how team resilience assists team overcome obstacles during a creative process. According to the study, everyone in a group may grow more robust if it can do more inventive work [42].

Therefore, it is necessary to accept the claim that school support can generate a good impact on the relationship amongst resilience of teachers and initial teaching performances. Or to put it another way, innovative teaching strategies should benefit from school assistance. So, according to [43], school support produced a constructive impact on innovative teaching process. This illustrates how resilience improves a person's and kids' capacity for adaptability and creativity. Researchers have emphasized the value of resilience teaching in the learning environment that can assist in indirectly lowering dropout rates and absenteeism [44].

According to data, persons with insufficient levels of resilience and coping abilities are more likely to suffer negative mental and psychological impacts during stressful events (e.g., disaster, tragedy, and disease outbreak) [39]. Resilience is important for individuals to bounce back and be able to adjust. Support provided by, coworkers, families, peers, and classmates can assist people maintain emotional stability in the face of danger and stressful situations [45].

According to the most recent research [39, 46, 47], students with strong trait resilience show a constructive link amongst creative thinking and PTSS, demonstrating the moderator influence of trait resilience in this relationship. According to the findings, increasing creative thinking may be a sign of post-traumatic development in the cognitive process [48]. More mentally resilient individuals are more creative than less emotionally resilient individuals [16]. Resilience practice should be implemented in educational settings since the researchers in the studies described above have observed the emotional changes brought on by resilience creativity and practice.

### Autonomy support

Being able to decide based on one's observations of the outside world is what is meant by autonomy. When learners are independent, they have control over their behavior because they can justify it with an internal authority [49]. To be competent, students must be capable to exercise and use their skills in routine life as well as be influential in their continual communication with the cultural environment. External variables provide feedback on a person's talents or competence as well as encouragement for competency [50]. Learners who engage more in autonomous learning can develop in environments where they feel comfortable as members of a community [51, 52].

The endeavor to deliver teaching in a class setting that advocates students’ needs for autonomy and the interaction amongst students and educators is related to the autonomy support outlined by [53]. To be clear, educators' actions and attitudes are crucial components that may be utilized to identify, enhance, and grow learners' innate motivating talents. According to [53], the main goal of autonomy support is to reaffirm and make clear that the teaching method, class environment, and relationship amongst teacher and student are all conducive to enhancing autonomy.

Behaviors that support freedom of choice include listening to students’ opinions and offering a range of instructional options, developing their motivating abilities, welcoming their perspectives, outlining how activities may be completed, and speaking to them in an unobtrusive manner [54]. Academic achievement, enthusiasm to study, and engagement in the classroom all increase when autonomy is encouraged by the instructor [55]. Their motivation and participation in the classroom are raised by meeting their requirements. As a result, individuals are more probably to have better physical and mental health and do better in school [56].

According to [5], the learners in autonomous learning should be motivated to learn by making a good first impression, and the content offered to them should be appropriate for their level of proficiency. However [36], described a learning setting in which the ideas of the students were voiced in their study. The majority of the respondents in their examination claimed that in several classes, when they uttered their feelings on the materials, the course purposes, the activity types, or the allotted time, they were not voiced. The students are treated as active participants, much as in autonomous learning, and their interests and requirements are given first importance. Many of the pupils acknowledged that they had to work in groups during a few courses, and they clarified that this was a good experience since they could exchange information and use the time together to concentrate on particular subjects. In addition [57], stated that learner autonomy was seen to be necessary for the group work activities.

According to [58], instead of attempting to educate learners how to be autonomous, we should provide them opportunity to do so. The establishment of learner autonomy must be a continuous process including social interactions with instructors and peers. By building on their prior knowledge, learners may maintain their autonomy throughout this process. Using the SCT lens [59], claims that autonomous activities help students develop accountability for the continuing mediation, increase their ZPD, and overall enhance autonomy of learning, which entails instantaneous dependency and independence. Scaffolding offers students the chance to benefit from social relations with the assistance of or cooperation with more experienced classmates, professors, and members of a community as an autonomous activity. According to [60], teaching scaffolds enhances students' reading comprehension abilities and is crucial in encouraging reading for autonomous understanding. Further research is required to determine how scaffolding affects EFL students’ English learning in groups and how it might increase student autonomy [61].

### Task-supported language teaching (TSLT)

A task is described as an instructional work-plan in task-based language teaching (TBLT) that emphasizes the authenticity of the target language and engages students in utilizing and processing the language practically while paying heed to grammar rules to reach an upshot that is situationally appropriate [62]. A powerful TBLT framework is students-centered in that student’s 'notice' language rules while engaging in communication activities, contrary to the conventional teacher-oriented presentation-practice-production (PPP) strategy [63]. A weak TBLT version, also identified as task-supported language teaching (TSLT), on the other hand, employs a structured syllabus but regards tasks as important components of a pedagogical environment [64].

This TSLT technique is consistent with the conventional PPP strategy that bases production on tasks and offers students language-driven learning communications and activities that enable real-world language output, the identification of linguistic deficiencies, and the receipt of corrective feedbacks [64]. In the past two decades, communicative approaches have been introduced to EFL nations as a central syllabus novelty to substitute the conventional grammar-translation approach [65]. However, many implementation attempts of communicative approaches have been unsuccessful. Large sizes of the classes, weak management of the classrooms, lack of teaching aids, insufficient communication resources, and the communication of these methods with the native cultures, instructors’ cognition, the present testing system, and pupils’ prior learning capabilities are all potential issues [66]. The development of high school EFL students' communication skills in Saudi Arabia must take into account the cultural context of Saudi Arabia for English instruction. Therefore, TSLT is considered in the current study to be a more appropriate instructional strategy than TBLT [67].

The design, implementation, and use of TBLT theories by teachers in various FL contexts, as well as how learners intended to prepare assignments, have all been the subject of an increasing number of papers and empirical investigations in recent years [68]. The study linking TSLT and its impact on EFL learners' autonomy, resilience, and creativity is still few, despite the fact that many TBLT investigations were performed on the learning of English abilities and sub-skills.

### Empirical studies

Some empirical studies confirmed the positive effects of tasks and SA on language learning, for example [69], examined how task-supported feedbacks affected the fluency, accuracy, and organization of 72 Iranian students. Three groups were included in this research: a control group that received no tasks; a task-supported group; and a task-supported group that received interactive feedbacks. The research results showed the task-supported group outdid the other groups on the post-tests.

In another research [70], looked researched the impact of TBLT on 52 starting level Mandarin learners' motivation to study as well as the related elements influencing that motivation. A quantitative questionnaire was administered and to identify the variables influencing learning motivation, semi-structured interviews were performed with 11 distinct grade-based students out of a total of 52 learners in the three survey stages. The results showed that learning motivation increased statistically between the first cycle post-treatment survey and the second cycle post-treatment survey whereas there was a non-statistically substantial decrease amongst the pre-treatment survey and the first cycle post-treatment survey. The consequences of the interview depicted that the use of TBLT, a busy schedule, and assistance from Mandarin native speakers all had an impact on the learners' willingness to study. The study's findings concluded that TBLT might boost students' motivation to learn over the long run.

Moreover [71], looked at how supporting activities, SA, and peer-assessment all worked together to affect test-taking abilities in online assessments. The study looked at how combining these three factors improved self-, peer-, and critical thinking during online assessments. The consequences exhibited that supporting activities, SA, and peer-assessment all worked together to improve test-taking abilities in online exams. The combination of these factors encouraged critical thinking, self- and peer-evaluation, which boosted performance and deepened topic learning.

In one more research [72], looked at how supporting tasks affected respondents' self-reports during online tests. Undergraduate people took part in the research, and they received supporting activities to help them with their SA process, including rubrics and instructions. The findings showed that the addition of supporting activities considerably increased students' accuracy in self-evaluation and their capacity to pinpoint areas that needed development. The results indicated that adding supporting tasks could improve the efficiency of SA in online tests.

Also [73], sought to understand more about how Iranian EFL students' metacognitive awareness and autonomy were affected by writing self- and peer-assessment. Utilizing convenience sampling, 120 individuals were chosen. In this investigation, a quasi-experimental approach was employed. They were split into a control group of 40 people, a self- and peer-assessment group of 40, and two experimental groups. ANCOVA was used to test and compare the data that had been collected. The tests' results showed that SA and peer-assessment were both useful strategies for enhancing EFL students' autonomy and metacognition awareness when completing writing assignments.

Additionally [74], looked at the effects of learners’ autonomy and its components, as well as language competency. Additionally, it sought to determine how the students felt about using the strategy. To achieve the goals, a control group (25 participants) and an experimental group (24 participants) were selected. The identical versions of PET were used to examine student proficiency in the pretest and posttest. By utilizing a multidimensional learner autonomy scale, the degree of student autonomy was also investigated during both the pre-test and post-test. Over the course of 25 sessions spread over three months, SA was used. There was no effect of the approach on language competency, according to a t-test study of the outcomes of the post-test proficiency exam. Even though only three of the questionnaire's dimensions—those used to evaluate the data—showed improvement, there was evidence of improvement in learners’ autonomy overall.

Furthermore, the influence of students' learning autonomy and SA on their performance in vocational education was examined by [75]. With the help of the random-matching sample procedure, sixty pupils were chosen. SA and performance test rubrics were employed as the study tools during the pre and post-tests phases of the data collection process. With the aid of SPSS.25, the gathered information were quantitatively evaluated and the findings indicated that students' performance was influenced by their own evaluations as well as their level of learning autonomy. Students' performance increased as a result of SA, which was directly influenced by learning autonomy. Additionally, the outcomes demonstrated that pupils were actively involved in the process of learning and their learning autonomy had the greatest impact on their ability to evaluate their own performance. However, their counterparts in the task group obtained better scores in the writing section of the test.

The related literature indicates that applying SA in EFL classes have generated positive effects on language learning and teaching. Also, the literature shows that EFL learners need to be more autonomous in language learning and appropriate settings should be provided for them to become more creative. Additionally, based on the literature, there are few studies on the effectiveness of SA on Saudi Arabian EFL learners’ autonomy, resilience, and creativity. Therefore, this research attempted to cover the gap by posing the following questions and hypotheses:

In this research, three questions and three hypotheses were formed:

- RQ1. Is Saudi Arabian EFL learners’ autonomy developed by practicing self-assessment in task supported language learning?
- RQ2. Is Saudi Arabian EFL learners’ creativity developed by practicing self-assessment in task supported language learning?
- RQ3. Is Saudi Arabian EFL learners’ resilience developed by practicing self-assessment in task supported language learning?
- HO1. Saudi Arabian EFL learners’ autonomy is not developed by practicing self-assessment in task supported language learning.
- HO2. Saudi Arabian EFL learners’ creativity is not developed by practicing self-assessment in task supported language learning.
- HO3. Saudi Arabian EFL learners’ resilience is not developed by practicing self-assessment in task supported language learning.

## Methodology

### Research design

A quasi-experimental design containing pre-test- treatment- post-test with non-random accessibility sampling for choosing the participants was used in this investigation. One CG and one EG formed the participants of this research. Practicing SA in task supported language learning was the independent variable and resilience, creativity, and autonomy were the dependent variables in this research.

### Participants

Using the purposive sampling method, a sample of 60 Saudi Arabian students was chosen from the Prince Sattam Bin Abdulaziz University in Al-Kharj, Saudi Arabia. These were all intermediate-level pupils, ranging in age from 17 to 29. In fact, it was made sure that the research subjects had a particular degree of written and spoken English competence. All of the subjects were males and they were split into two groups: experimental and control.

### Research instruments

The OQPT was the first tool exploited in the present experiment to homogenize the subjects. It assisted the researchers in choosing the same subjects. The students considered to be the intermediate and the study's responses were those who scored between 40 and 47 on the test's 60 multiple-choice questions.

To assess the students' autonomy, a learner autonomy scale was also employed as a pretest and posttest. Forty-four statements based on nine language learning-related aspects were included in the questionnaire. The nine dimensions' elements indicate whether students demonstrate stronger levels of control over a certain area of their learning. The LAQ was chosen for this study because, according to [76] and [77], it was the most complete regarding the number of dimensions and, consequently, content validity comparing to other scales accessible in this domain. After administering the exam to 20 students as a pilot, and based on the suggestions of the experts, several questions were changed or eliminated in order to better fit the Saudi Arabian context.

The multi-dimensional construct measure created [78] was adopted as the academic resilience scale utilized in this study. It has one vignette and a questionnaire with 30 items on a five-point Likert scale (from 1. Totally agree to 5. Totally disagree). The vignette aided students in conceiving of themselves as facing academic difficulty, and the ARS-30 then examined their reactions to this condition [78]. Each participant spent between 20 and 30 min to complete the questionnaire. The KR-21 formula was employed to determine the instrument's reliability (*r* = 0.88), and several professors of applied linguistics from Tehran University verified the instrument's validity.

The Torrance Test of Creative Thinking (TTCT) [79] was used to assess the individuals' levels of creativity. This exam has been used extensively in several educational research, and according to [80], its reliability is 0.80. The test comprised of 60 items, each followed by three responses that represent various fictitious scenarios for the participants to answer to. The sum of the total results is classed as "low = up to 75; mid = 76–85; high = 86–120" based on the test's own scoring scale. In data coding, creative levels were denoted by a 0 for low, a 1 for medium, and a 2 for high. This test was scheduled to take place for 30 min. The reliability of the survey was assessed by means of Cronbach Alpha (*r* = 0.85), and it was verified by a committee of English specialists. It should be emphasized that both of the aforementioned questionnaires served as the study's pre- and post-tests; they were given before and after the intervention.

### Research procedure

Sixty Saudi Arabian EFL learners from Prince Sattam Bin Abdulaziz University in Al-Kharj, Saudi Arabia, were selected for the research population. They were then split into two groups—a CG and an EG—at random. Three related questionnaires were used to pre-test them on their capacity for resilience, creativity, and autonomy. Then, the treatment was practiced on the two groups. Regarding the method of treatment, the EG received the treatment by using SA in a task supported language learning. Eight lessons (vocabulary, grammar, and reading comprehension) of Touchstone book 3 were trained to this group using SA via applying different tasks. On the other hand, the lessons were trained to the participants in the CG traditionally; without using SA and tasks. Under the direction of the researchers, the treatment lasted for 24 sessions of 50 min each. The OQPT was given to the students after the goals and methods of the study were outlined to them in the first session. The group members took three sessions of pre-testing. The treatment was used over the course of 17 sessions. The post-tests were taken by the groups during the final three sessions. The collected data were then appropriately examined.

### Analyzing the data

For analysing the collected data, SPSS software, version 22, was utilized. First, the quality of data normality was assessed using the Kolmogorov–Smirnov (K-S) test. In the second step, descriptive statistics were computed. Third, to determine the influences of the treatment on the pupils’ resilience, creativity, and autonomy, independent and paired samples t-tests were conducted.

## Findings

The required data were gathered, and then they were analyzed to attain the results. The results of the K-S test proved the normality distribution of the data as all Sig. values were greater than 0.05. Subsequently, the parametric statistics including paired and independent samples t-tests were performed to analyse the data in order to gain the ultimate results.

As displayed in Table 1, the average of the control students is 58.66 and the average of the experiment class is 59.96 on the creativity pre-test. Based on averages, both classes possessed equal level of creativity at the commencement of the study.

**Table 1:** Descriptive statistics of the creativity pre-tests

Groups	N	Means	Std. Deviations	Std. Error Means
CG	30	58.66	7.93	1.44
EG	30	59.96	6.13	1.12

Table 2 shows the results of an independent samples t-test to see if there were significant variations in the two groups' results on the creativity pre-tests. As Sig (0.48) is larger than 0.05, the difference between the groups is not statistically significant at the commencement of the study.

**Table 2:** Inferential statistics of the creativity pre-tests

		Levene's Test for Equality of Variances		t-test for Equality of Means				
		F	Sig	t	Df	Sig. (2-tailed)	Mean Difference	Std. Error Difference
Scores	Equal variances assumed	1.68	.20	-.71	58	.48	-1.30	1.83
	Equal variances not assumed			-.71	57.55	.48	-1.30	1.83

The above table (Table 3) exhibits the descriptive statistics of the students on the creativity post-tests. They had different performances on the creativity post-tests as the mean score of experimental class (M = 67.73) is greater than the control class (M = 61.16).

**Table 3:** Descriptive statistics of the creativity post-tests

Groups	N	Means	Std. Deviations	Std. Error Means
CG	30	61.16	6.80	1.24
EG	30	67.73	16.41	2.99

As Table 4 displayed, there is a substantial variation amongst the two groups' creativity post-tests at (*p* < 0.05). Truly, the experimental class obtained better accomplishment than the control class on the post-test of creativity.

**Table 4:** Inferential statistics of the creativity post-tests

		Levene's Test for Equality of Variances		t-test for Equality of Means				
		F	Sig	t	Df	Sig. (2-tailed)	Mean Difference	Std. Error Difference
Scores	Equal variances assumed	12.09	.00	-2.02	58	.04	-6.56	3.24
	Equal variances not assumed			-2.02	58	.05	-6.56	3.24

The creativity pre- and post-tests of each class are compared via a paired samples t-test in Table 5. The creativity pre-test and post-test differences in the control class are substantial as Sig (0.03) is lower than 0.05, and likewise, the creativity pre-test and post-test differences in the experimental group are meaningful because Sig (0.00) is less than 0.05.

**Table 5:** Paired samples test of the creativity pre and post–tests

		Mean	Std. Deviation	Std. Error Mean	t	df	Sig. (2-tailed)
Pair 1	CG pre – CG post	-2.96	7.33	1.31	-2.25	29	.03
Pair 2	EG pre – EG post	-7.70	14.06	2.52	-3.05	29	.00

Based on the data in Table 6, since the two groups' mean scores were very close, we may conclude that they conducted similarly on the resilience pre-tests, as shown by the descriptive data in Table 6. Based on the descriptive data, the mean score of CG is 75.33 and the mean score of the EG is 74.39, this means that both groups had similar level of resilience before the treatment.

**Table 6:** Descriptive statistics of the resilience pre-tests

Groups	N	Means	Std. Deviations	Std. Error Means
CG	30	75.33	8.62	1.57
EG	30	74.39	8.66	1.58

Table 7 exhibits that Sig (0.65) is bigger than 0.05, subsequently, the difference amongst the control and experimental students is not meaningful. Accordingly, the resilience pre-tests of the control and experimental classes do not vary meaningfully from one another.

**Table 7:** Inferential statistics of the resilience pre-tests

		Levene's Test for Equality of Variances		t-test for Equality of Means				
		F	Sig	t	Df	Sig. (2-tailed)	Mean Difference	Std. Error Difference
Scores	Equal variances assumed	.00	.96	.44	58	.65	1.00	2.23
	Equal variances not assumed			.44	57.99	.65	1.00	2.23

Table 8 reveals the two classes’ descriptive statistics on the creativity post-tests. They had dissimilar performances on the creativity post-tests as the average of the experimental class (M = 107.70) is greater than the control class (M = 77.80).

**Table 8:** Descriptive statistics of the resilience post-tests

Groups	N	Means	Std. Deviations	Std. Error Means
CG	30	77.80	9.86	1.80
EG	30	107.70	13.54	2.47

The difference between control and experimental classes is obviously apparent in Table 9 as Sig (0.00) is lower than 0.05. This means that the creativity post-test outcomes of the experimental students are remarkably dissimilar to those of the control students.

**Table 9:** Inferential statistics of the resilience post-tests

		Levene's Test for Equality of Variances		t-test for Equality of Means				
		F	Sig	t	Df	Sig. (2-tailed)	Mean Difference	Std. Error Difference
Scores	Equal variances assumed	8.08	.00	-9.77	58	.00	-29.90	3.05
	Equal variances not assumed			-9.77	58	.00	-29.90	3.05

Table 10 reveals that the difference amongst the creativity pre-test and creativity post-test of the control class is outstanding because Sig (0.02) is less than 0.05, they are also meaningful for the experimental students as Sig (0.00) is lower than 0.05.

**Table 10:** Paired samples test of resilience pre and post—tests

		Mean	Std. Deviation	Std. Error Mean	t	df	Sig. (2-tailed)
Pair 1	CG pre – CG post	-2.93	6.85	1.23	-2.25	29	.03
Pair 2	EG pre – EG post	-32.48	10.19	1.83	-3.05	29	.00

The descriptive characteristics for the two groups are presented in Table 11. The mean score for the experimental group is 101.56 whereas the mean score for the control group is 99.83. This suggests that the degree of autonomy in the two groups was equivalent at the outset of the research.

**Table 11:** Descriptive statistics of the autonomy pre-tests

Groups	N	Means	Std. Deviations	Std. Error Means
CG	30	99.83	16.71	3.05
EG	30	101.56	15.20	2.77

On Table 12, an independent samples t-test was run to show the differences between the two groups' autonomy pre-test scores. The results show that the Sig value (0.67) is more than 0.05, indicating that the differences between the groups are not statistically significant. On the autonomy pretest, they really conducted similarly.

**Table 12:** Inferential statistics of the autonomy pre-tests

		Levene's Test for Equality of Variances		t-test for Equality of Means				
		F	Sig	t	Df	Sig. (2-tailed)	Mean Difference	Std. Error Difference
Scores	Equal variances assumed	.58	.44	-.42	58	.67	-1.73	4.12
	Equal variances not assumed			-.42	57.48	.67	-1.73	4.12

According to the descriptive data in Table 13, the control group's mean score on the autonomy post-test was 103.91, while the experimental group's mean score was 141.76. On the autonomy post-tests, the experimental group seemingly outdid the control students.

**Table 13:** Descriptive statistics of the autonomy post-tests

	Groups	N	Means	Std. Deviations	Std. Error Means
Scores	CG	30	103.91	19.00	3.46
	EG	30	141.76	16.74	3.05

According to Table 14, the difference between the experimental and control participants is statistically remarkable. In actuality, on the autonomy post-test, the experimental group outstripped the control group. Since the Sig value (0.00) is lower than 0.05, there exists a meaningful difference amongst the performances of both classes on the autonomy post-tests in favor of the experimental class.

**Table 14:** Inferential statistics of the autonomy post-tests

		Levene's Test for Equality of Variances		t-test for Equality of Means				
		F	Sig	t	Df	Sig. (2-tailed)	Mean Difference	Std. Error Difference
Scores	Equal variances assumed	2.14	.14	-8.19	58	.00	-37.86	4.62
	Equal variances not assumed			-8.19	57.09	.00	-37.86	4.62

In Table 15, the pre- and post-test results for each group are compared using a paired samples t-test. Because the Sig value (0.00) is less than 0.05, there is a difference in the control group's performance before and after the treatment. Similarly, the experimental group's pre- and post-test differences are notable because the Sig value (0.00) is less than 0.05.

**Table 15:** Paired samples test of autonomy pre and post—tests

		Mean	Std. Deviation	Std. Error Mean	t	df	Sig. (2-tailed)
Pair 1	CG pre – CG post	-4.48	8.31	1.49	-3.00	29	.00
Pair 2	EG pre – EG post	-39.09	16.18	2.90	-13.44	29	.00

In short, the results of this study indicate that both groups were homogenous in terms of English language resilience, creativity, and autonomy before receiving the treatment. After the treatment, the performances of the two groups were different; implying that the EG outflanked the CG on the post-tests of resilience, creativity, and autonomy.

## Discussion, conclusion, and implication

A meaningful difference between the post-tests of the control and experimental classes were shown by the obtained results. In actuality, the experimental group outperformed the control group because they scored higher on their post-tests. This improvement can be linked to the care they got through task-supported language learning that included SA.

Sadeghi K. et al. [81] verified the good impacts of task-based orientated activities on enhancing grammar recognition in EFL learners, supporting the results obtained. Additionally, our results are consistent with the survey of [69], who looked at the impact of task-supported interactive feedback on the fluency, accuracy, and organization of EFL students. They demonstrated that the task-supported group did better than the control group in all three writing-related areas. Additionally, [70], who confirmed the beneficial effects of TBLT on 52 Mandarin learners' motivation to study English, concur with our findings. Our findings are further corroborated by [82], who show how task-supported learning implementation affects students' empowerment.

Additionally, the results of [73] concur with our findings since they showed that EFL students' metacognitive consciousness and autonomy were positively affected by self- and peer assessment. Furthermore, our findings concur with those of [74], who claimed that employing SA had a good effect on boosting the autonomy of EFL students. Additionally, the conclusions of the existing study are along with those of [75], who asserted that SA increased students' learning autonomy.

Our consequences are in accord with those of [83] who defended the importance of SA in developing the writing skill of EFL pupils. The results of the current study are consistent with those of [84], who discovered that SA improved EFL pupils’ writing skills. Our findings are supported by [85] comparison of the impact of self- and peer-assessment on EFL students’ oral ability. His research revealed that oral ability of EFL learners was equally impacted by peer-assessment and SA.

Our results are supported by the theory of situated language learning theory stating that learning a language is more efficient if it is conducted in a community, where the target language is used in real context and for real tasks. It aids in capturing the authentic context where learners are immersed in the natural and meaningful milieu spontaneous [86]. In addition, our study is supported by constructivist theory which advocates the full engagement of learners in the construction of their own knowledge. In order to construct new sound knowledge, learners must assess this knowledge to fill gaps in it and to make sure of connections between its parts.

SA is a crucial part of constructivist and cognitive theories of learning and motivation. [87] made the point that the knowledge production at the core of such theory depends on student self-monitoring of their learning and thinking. In other words, prior to and during learning, students generate meaning in part through self-evaluation. Self-evaluation is a step in the organizing, evaluating, and internalizing steps that students go through during learning. They must make connections between new information, insights, and abilities and what they have already learned and applied. SA helps pupils develop their capacity to draw these conclusions for themselves, offers a way to make learning more meaningful than rote, and boosts students' motivation and self-assurance.

The benefits of task-oriented learning, where learners are at the center of learning and work on something that is personal and important to them, can be credited for the experimental group's improved performance. Learners pass a lot of time talking and getting practice working in groups to make choices. Additionally, task-based learning emphasizes involving students in worthwhile tasks that call for them to utilize the target language. Instead of learning grammatical rules and vocabulary lists by heart, language learners exercise their skills in real-world situations where they can communicate.

Task-based learning has numerous benefits over traditional teaching techniques, including motivating students by making the language more engaging and relevant and by giving them chances for real-world involvement. Task-based learning also promotes creativity and autonomy in learners, combines the four skills and many facets of language in a holistic fashion, and exposes learners to a variety of genres, registers, and language usage styles.

In a TBL atmosphere, students often participate actively and with high motivation in assignments and activities. It provides a platform for students to demonstrate their abilities and boost their development. As they collaborate and work together in groups, language learners build ties with one another. They can demonstrate and develop meaningful engagement on a particular subject when working in groups. Additionally, the class collaborates and evaluates the overall success of the course. Students draw on prior language, knowledge, and experience during all three phases of a task-based learning session rather than focusing on just one component of a particular language characteristic. The pupils are able to investigate both old and new linguistic aspects thanks to this method. According to [88], task-based learning places an emphasis on encouraging students to interact with one another in the target language, brings genuine texts into the classroom, inspires pupils to pay notice to both the language and the learning process, and incorporates the students' own personal experiences as significant teaching resources.

Another explanation for the experimental group's improved performance is the benefit of SA, which can foster the abilities of reflective practice and self-monitoring. Through self-reporting of learning progress by students, it can encourage academic honesty and foster independent learning. SA can boost student motivation and aid in the development of a variety of unique and transferable abilities. Students are able to evaluate their own performance through SA. It may be incredibly beneficial in fostering students' capacity for self-reflection, criticism, and judgment. In the end, it teaches students how to take ownership of their own education.

Using SA which pushed learners to study autonomously to accomplish their learning purposes and improve their capabilities for future performance, may have contributed to the experimental group's success on the posttests of resilience, creativity, and autonomy. By allowing them to gauge how much they have learned, SA motivates pupils to advance and succeed in fulfilling all criteria, claim [89]. Students may be inspired to assume more responsibility for improving their performance via SA. This is significant because [90], who support it, claim that SA enables students to be somewhat aware of their accountability toward the learning objectives in terms of learners’ speaking abilities.

Providing opportunities for learners to reflect on their work and processes is a powerful way to develop the learning journey. SA and self-reflection involve students reviewing their work and reflecting on their learning progress. This helps students participate in and take ownership of their own learning. SA and self-reflection is a useful way to improve a student’s learning experience. It plays a vital role in teaching students not just what to learn, but also how they learn and what they can do to enhance their learning outcomes. By integrating tasks that require students to critically reflect on their work, processes and learning style; they are given the opportunity to identify gaps in their knowledge or skill set and achieve greater autonomy and deeper learning and metacognition. The mentioned positive features of SA can be the reasons of the gained results in the present study.

One more reason for the gained results can be attributed the nature of task-based learning; task-based learning is conducive to group learning. Learning a language as a group is also a very important contributor to effective retention. Collaborating with others and becoming confident with the language within a group is a key step in acquiring that language. Also, receiving positive feedback from peers and teachers increases confidence and motivation to learn and to communicate with others.

In a word, the outcomes of this investigation depict that SA may considerably increase Saudi Arabian EFL learners' resilience, creativity, and autonomy. The use of more pedagogical techniques like SA and activities in language classes is thus strongly suggested, especially for those who concentrate on psychological difficulties. We may infer that pupils become more self-directed and motivated learners when they participate in evaluating their own performance. Students can also gain metacognitive abilities through SA, which help them to reflect on their learning experience, pinpoint areas for growth, and create reasonable objectives. In addition to improving their linguistic abilities, this gives students practical skills for everyday living. It may be inferred that a variety of factors may influence how well learners pick up new languages. One of them may be self-evaluation. With SA, students take an active part in the evaluation process and contribute to the advancement of language acquisition. By helping students evaluate their own achievements objectively, SA promotes lifelong learning.

The results of this study may have some implications for EFL instructors, students, and curriculum developers. Using SA can allow teachers to delve into specific aspects of their teaching practice. Also, it aids teachers to create critical reflective practice in their own actions. Teachers who engage in SA have a more insightful understanding of the content, students’ needs, and the pedagogical knowledge required to teach effectively. SA helps classroom teachers become more aware of which metacognitive tactics to employ and when. When educators create clear learning objectives and provide evaluation standards that permit pupils to evaluate their own work, both students and teachers acquire these abilities. Through active participation in the learning process and increased connection to and commitment to the learning goals, these approaches engage learners. By scaffolding and demonstrating reflection, goal-setting, strategy adjustment, and evaluation instructors must learn to help students take on evaluative duties. Scaffolding, which aims to give students more responsibility, necessitates that professors take a back seat and act as coaches and consultants while students gain knowledge from their own experiences [91]. Students are more motivated and engaged when they have confidence in their ability to finish a task. Therefore, while students set objectives and go through their self-evaluations, teachers should continue to have high expectations for their students' success. In this approach, student SA in the classroom creates distinct learning objectives, specifies evaluation standards, offers resources for assessment, and gives students time for reflection.

Students may be inspired to critically measure their own learning progress and performance as a result of the research's consequences. Additionally, the ramifications may inspire students to take more ownership of their education. Students' ability to make judgments is improved by self-evaluation. Students who evaluate themselves are not subject to peer pressure. Students who self-assess can study independently and become more self-aware of their strengths and weaknesses. By using SA, students can objectively reflect on and critically evaluate their own progress and skill development. Also, they can identify gaps in their understanding and capabilities and discern how to improve their performance. Furfure, the results of this research can help students to learn independently and think critically. SA can also help in keeping track of students’ progress, which will help them to retain the information needed. In addition to using a variety of assessments, teachers may encourage students' autonomy in the classroom by having them participate in SA activities. As a result, EFL students may gradually comprehend what it takes to become self-directed learners. Students may also identify precisely where they need help and support by completing SA assignments, allowing them to ask their instructors for assistance.

The research outcomes also benefits syllabus and material creators. The results may assist syllabus designers have a clearer comprehension of SA and how it may influence EFL students’ language acquisition. They are suggested to include SA exercises in their curricula as SA has been confirmed to be helpful in enhancing EFL learners' language acquisition. The results of this study may also help material designers develop a range of tasks and activities that are suitable for EFL students' English proficiency levels and sub-skills.

Further research can use a qualitative research design; this survey utilized a quantitative research strategy. In addition to the surveys, other tools like observation and interviews should be used to acquire a full picture of the students' perspectives and to provide more accurate findings. Additionally, only intermediate-level EFL students took part in the investigation; beginning and advanced pupils were not taken into account. As a result, next investigations are required to account for the influence of these variables. Additionally, the researchers of this study solely looked at how Saudi Arabian EFL learners' autonomy, resilience, and SA skills were affected. Conducting research on how SA affects other language abilities and sub-skills is fascinating. Examining the function of the task-based method in language testing would be one of the future avenues for this field of study. The effects of various task types on students’ language competence and psychological variables might also be the topic of future research.

## Data Availability

The dataset of the present study is available upon request from the corresponding author.
